# E-Cadherin Destabilization Accounts for the Pathogenicity of Missense Mutations in Hereditary Diffuse Gastric Cancer

**DOI:** 10.1371/journal.pone.0033783

**Published:** 2012-03-21

**Authors:** Joana Simões-Correia, Joana Figueiredo, Rui Lopes, François Stricher, Carla Oliveira, Luis Serrano, Raquel Seruca

**Affiliations:** 1 IPATIMUP - Institute of Molecular Pathology and Immunology of the University of Porto, Porto, Portugal; 2 Center of Ophthalmology and Vision Sciences, IBILI - Institute for Biomedical Research in Light and Image, Faculty of Medicine, University of Coimbra, Coimbra, Portugal; 3 Medical Faculty of the University of Porto, Porto, Portugal; 4 EMBL/CRG Systems Biology Research Unit, Centre for Genomic Regulation (CRG), UPF, Barcelona, Spain; 5 ICREA, Institució Catalana de Recerca i Estudis Avançats, Barcelona, Spain; National Cancer Center, Japan

## Abstract

E-cadherin is critical for the maintenance of tissue architecture due to its role in cell-cell adhesion. E-cadherin mutations are the genetic cause of Hereditary Diffuse Gastric Cancer (HDGC) and missense mutations represent a clinical burden, due to the uncertainty of their pathogenic role. In vitro and in vivo, most mutations lead to loss-of-function, although the causal factor is unknown for the majority. We hypothesized that destabilization could account for the pathogenicity of E-cadherin missense mutations in HDGC, and tested our hypothesis using in silico and in vitro tools. FoldX algorithm was used to calculate the impact of each mutation in E-cadherin native-state stability, and the analysis was complemented with evolutionary conservation, by SIFT. Interestingly, HDGC patients harbouring germline E-cadherin destabilizing mutants present a younger age at diagnosis or death, suggesting that the loss of native-state stability of E-cadherin accounts for the disease phenotype. To elucidate the biological relevance of E-cadherin destabilization in HDGC, we investigated a group of newly identified HDGC-associated mutations (E185V, S232C and L583R), of which L583R is predicted to be destabilizing. We show that this mutation is not functional in vitro, exhibits shorter half-life and is unable to mature, due to premature proteasome-dependent degradation, a phenotype reverted by stabilization with the artificial mutation L583I (structurally tolerated). Herein we report E-cadherin structural models suitable to predict the impact of the majority of cancer-associated missense mutations and we show that E-cadherin destabilization leads to loss-of-function in vitro and increased pathogenicity in vivo.

## Introduction

E-cadherin is a cell-cell adhesion glycoprotein comprised of five extracellular cadherin-type repeats, one transmembrane region and a highly conserved cytoplasmic tail [1], [2]. E-cadherin is expressed primarily in epithelial cells and is the major component of Adherens Junctions (AJ). These junctions cluster, via homophilic interactions, through the extracellular domains of calcium-dependent E-cadherin molecules, on the surface of homotypic neighbour cells.

The role of E-cadherin in tumour development is well described, and its loss of expression is a hallmark in carcinomas [3]. Experimental evidence supports a role for the E-cadherin complex both in suppressing invasion and metastasis formation [4]. Loss of E-cadherin expression is frequently associated to genetic events such as splice site and truncation mutations caused by insertions, deletions, and nonsense mutations, in addition to missense mutations [5]. In sporadic diffuse gastric cancer, alterations in the gene encoding E-cadherin (CDH1) are found preferentially in exons 7 to 9 [5], while in lobular breast cancers they are spread along the gene, with no preferential hotspot [6]. Missense mutations are found in these two types of sporadic cancer and also in synovial sarcomas [7].

Familial aggregation of Diffuse Gastric Cancer (DGC) represents 10% of the cases of Gastric Cancer (GC), and only 1–3% are hereditary [8]. From these familial cases, Hereditary Diffuse Gastric Cancer (HDGC) is defined by stringent criteria that were defined by the International Gastric Cancer Linkage Consortium (IGCLC) in 1999: (1) two or more documented cases of diffuse gastric cancer in first/second degree relatives, with at least one diagnosed before the age of 50; or (2) three or more cases of documented diffuse gastric cancer in first/second degree relatives, independently of age. Early Onset Diffuse Gastric Cancer (EODGC) is considered when an isolated individual is diagnosed with DGC with less then 45 years of age. Germline CDH1 mutations are found in 30% of the HDGC cases [9]. The association of CDH1 mutations and familial gastric cancer was first described by Guilford *et al* in 1998 [10] and since then many studies reported different types of CDH1 mutations in HDGC [11], [12], [13]. Among all reported *CDH1* germline mutations, 77.9% are nonsense, splice-site and frameshift mutations (predicted to produce premature termination codons) and 22.1% are missense mutations [9]. Mutations that generate PTC are normally deleterious, the patients are considered high risk carriers, and are advised to have prophylactic total gastrectomy [14]. The pathogenicity of missense mutations is not straightforward, and these alterations are commonly referred as Unclassified Sequence Variants (USVs) due to the lack of stringent criteria to evaluate their impact. Several parameters have been taken into account for the classification of E-cadherin USVs in HDGC: 1) co-segregation of the mutation with DGC (within pedigrees); 2) mutation frequency in the healthy control population; 3) mutation recurrence (in independent families). Segregation analysis is often impossible, with a small number of affected cases available for molecular diagnosis [15], and the absence of clinical information is a limiting step to infer the pathogenic significance of these mutations. To circumvent this limitation we have previously developed *in vitro* functional assays to evaluate the functional impact of E-cadherin germline missense mutations [16], [17]. However, such studies implicate lab specific experimental conditions, namely cell biology assays, and they are time consuming to use in routine. *In silico* predictions are reliable and fast analysis that one can use to predict the impact of point mutations, especially when structural information is available [18], [19].

In this work, we explored the potential of structure-based *in silico* predictions to evaluate the impact of E-cadherin missense mutations, found in hereditary and sporadic cancer. Our analysis was based on the calculation of native-state stability changes induced by each variant (ΔΔG = ΔG_WT_−ΔG_Mut_), obtained by the protein design FoldX algorithm [20], [21]. Interestingly, the group of patients harbouring destabilizing mutations (ΔΔG>0.8 kcal/mol) is characterized by a younger age at diagnosis or death by DGC, suggesting that the loss of E-cadherin native-state stability contributes to the disease phenotype. Using a cellular model, we analysed the phenotype of E-cadherin destabilization, and found that when a mutation induces decreased native-state stability, E-cadherin is prematurely degraded by the proteasome, exhibits shorter half-life, resulting in loss of the adhesive function. Altogether, our results suggest that destabilization accounts for the pathogenicity of E-cadherin missense mutations found in HDGC.

## Materials and Methods

### Collection of E-cadherin sequence variants and PDBs

E-cadherin variants associated to HDGC or EODGC were collected from the literature, and somatic variants were collected from the Catalogue of Somatic Mutations in Cancer (COSMIC) database (http://www.sanger.ac.uk/genetics/CGP/cosmic/). Three new E-cadherin sequence variants where reported to our lab for functional analysis: E185V, S232C and L583R. Recently, L583R was reported, with functional data associated [22].

E-cadherin-related PDBs were identified using automatic search with Swiss Model Repository (http://swissmodel.expasy.org). Sequence alignment of human E-cadherin and each of the sequences used for the different models was performed with M-coffee [23], [24] (http://tcoffee.crg.cat/apps/tcoffee/play?name=mcoffee). Images were prepared with Pymol. After analysing sequence and structural homology, three PDBs were selected to use as models: Xenopus C-cadherin ectodomain (PDB 1L3W), mouse E-cadherin prodomain (PDB 1OP4) and the mouse β-catenin interacting domain (PDB 1I7X).

### FoldX calculations and SIFT analysis

Using FoldX (http://foldx.crg.es/) command *Buildmodel* we built three different models (prodomain, extracellular and cytoplasmic); the three structures were humanized by substitution of each different aminoacid. The resulting structure was optimized using the command *RepairPDB* and the energies where analysed with *Stability* or *AnalyseComplex* commands. The disease-associated mutations were generated with the *Buildmodel* command, each mutation repeated in five runs. The energies are an automatic output in FoldX, and the native-state stability change, ΔΔG, between WT and mutant (ΔΔG = ΔG_WT_−ΔG_Mut_) is also generated in a separate file, with the corresponding standard deviations, and all the energetic penalties associated to each mutation. Only mutations with ΔΔG>0.8 kcal/mol were considered deleterious.

We used SIFT (http://sift.jcvi.org/, Sorting Intolerant From Tolerant) to evaluate the conservation of each aminoacid substitution, as previously described [25], using the Blink feature of GI: 31073. Only mutations with a score below 0,05 were considered to be Intolerant.

ProP (http://www.cbs.dtu.dk/services/ProP/) [26] was used to evaluate if mutations could have an effect on prodomain cleavage.

### Cell culture and transfections

E-cadherin WT cDNA was cloned in pIRES2-EGFP vector according to manufacture instructions (Clontech, Takara Bio) and mutations E185V, S232C, L583R and L583I hE-cadherin were induced by site directed mutagenesis as described previously [27]. The empty vector (Mock) was used as control.

CHO (Chinese Hamster Ovary) cells (ATCC number: CCL-61) were grown in Alfa-MEM medium (Gibco, Invitrogen) supplemented with 10% fetal bovine serum (FBS; Gibco, Invitrogen) and 1% penicillin-streptomycin (Gibco, Invitrogen). Cells were sporadically evaluated for mycoplasm contamination by imunofluorescence with DAPI. Cells were transfected with 1 ug of each of the vectors encoding the different forms of E-cadherin (WT, E185V, S232C, L583R and L583I) using Lipofectamine 2000 (Invitrogen), according to the manufacture procedure. For stable cell line establishment, cells were selected by antibiotic resistance to 5 µg/ml blasticidin (Gibco, Invitrogen). All cell lines were maintained in a humidified incubator with 5% CO_2_ at 37°C.

### Functional assays

Transiently transfected CHO (Chinese Hamster Ovary) cells (ATCC number: CCL-61) were subjected to flow citometry, using GFP fluorescence measurement, to evaluate the transfection efficiency before each experiment. For the slow aggregation assay, wells of 96-well-plate were coated with 50 µl of agar solution (100 mg Bacto-Agar in 15 ml of sterile PBS). Cells were detached with trypsin and suspended in culture medium. A suspension of 1×10^5^ cells/ml was prepared and 2×10^4^ cells were seeded in each well. The plate was incubated at 37°C in a humidified chamber with 5% CO_2_ for 48 h. Aggregation was evaluated in an inverted microscope (4× magnification) and photographed with a digital camera.

### Western blotting

Cell lysates were obtained with Catenin lysis buffer (1% Triton X-100, 1% Nonidet P-40 in PBS), supplemented with protease inhibitor cocktail (Roche) and phosphatase inhibitor cocktail (Sigma). Protein quantification was done by a modified Bradford assay (Bio-Rad). For each sample, 25 µg of protein was loaded, separated in 7.5% SDS-PAGE, and electroblotted to nitrocellulose membrane (GE Healthcare Life Sciences). Membranes were blocked with 5% non-fat dry milk and 0.5% Tween-20 in PBS. Immunoblotting was performed with antibodies against E-cadherin (1∶1000; BD Biosciences), actin (1∶1000; Santa Cruz Biotechnology), and α-tubulin (1∶10000; Sigma). Sheep anti-mouse (GE Healthcare Life Sciences) or donkey anti-goat (Santa Cruz Biotechnology) were used as secondary antibodies, followed by ECL detection (GE Healthcare Life Sciences). Immunoblots were quantified in Quantity One software (Bio-Rad).

### Fluorescence-activated cell sorting (FACS)

Cells were grown to a confluent monolayer, detached with Versene (Gibco, Invitrogen) and resuspended in ice cold PBS with 0.05 mg/ml CaCl_2_. A suspension of 5×10^5^ cells was centrifuged for 5 minutes at 1500 rpm 4°C, and washed in PBS with 0.05 mg/ml CaCl_2_ 3%BSA. Cells were incubated for 60 minutes with a primary antibody against E-cadherin, HECD1 (Zymed Laboratories) at 1∶100 dilution. Cells were washed twice and then incubated with anti-mouse biotinilated (Dako) at 1∶100 dilution. Cells were washed twice and then incubated with streptavidin PE-CY5 (BD Pharmingen) at 1∶40 dilution. Finally, cells were washed, resuspended in 500 µl of PBS, and 50000 cells were analyzed in a flow cytometer (Coulter Epics XL-MCL). Data was analyzed with WinMDI software.

### Immunofluorescent staining

For Immunofluorescence and microscopy, cells were seeded on glass coverslips and grown to about 80% confluence, fixed in ice-cold methanol for 15 minutes, washed 2 times with PBS, and incubated with primary antibody, diluted in PBS 5%BSA, for 60 minutes at room temperature. Primary antibodies used: mouse monoclonal anti-E-cadherin (BD Biosciences); rabbit anti-Calnexin (Stressgen). Secondary antibodies used: Alexa Fluor 488 anti-mouse (1∶500; Invitrogen); Alexa Fluor 594 anti-rabbit (1∶500; Invitrogen). The coverslips were mounted on glass slides, using Vectashield with DAPI for nuclear detection (Vector Laboratories). Image acquisition was performed on Carl Zeiss Apotome Axiovert 200 M Fluorescence Microscope using 40× objectives. Images were acquired with Axiocam HRm camera and processed by software Axiovison version 4.8.

### Cell Treatments

For the protein synthesis inhibition, cells were treated with 25 µM of Cycloheximide for 8 h and 16 h, and the amount of total E-cadherin was analyzed by WB as described previously. For the proteasome inhibition assay, cells were seeded in 6 well plates, grown to approximately 80% of confluence, and incubated for 16 h with 10 µM MG132 (CalBioChem). Cell lysates were analysed by WB as described previously.

## Results

### 1. E-cadherin structural models

**Figure 1:**
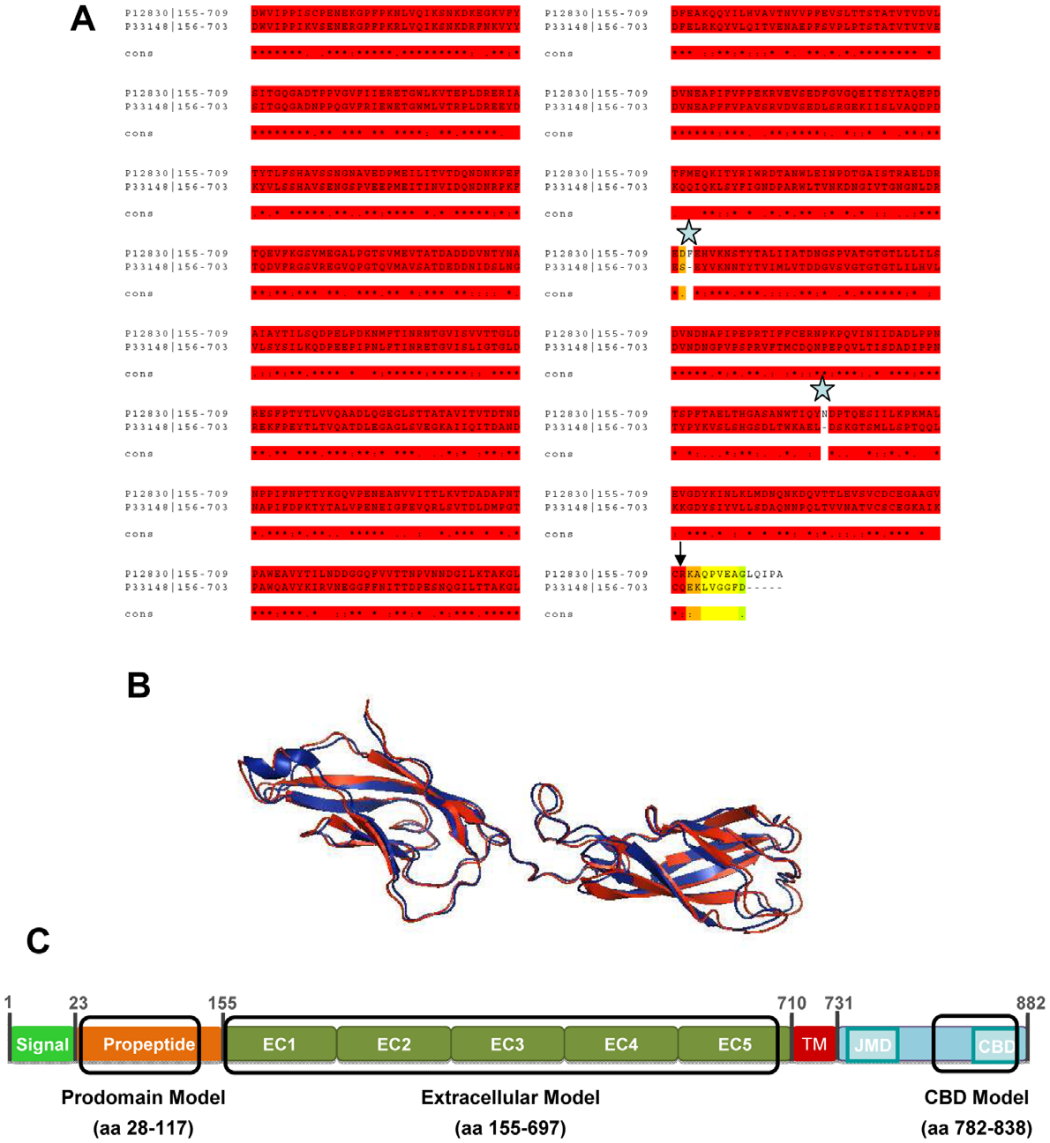
E-cadherin structural models. A) Sequence alignment of the extracellular domains of human E-cad and xenopus EP-cad. The extracellular sequences were obtained in Uniprot with the corresponding references (human E-cadherin, P12830; xenopus EP-cadherin, P33148). M-Coffee regular was used to perform the alignment, a package that combines different alignment methods. Red brick regions are in perfect agreement across all the methods, green and yellow regions are regions of no agreement between the different alignment methods. The average consistency score obtained was 98, confirming the reliability of the alignment. The blue stars identify the aminoacids that were removed from the 1L3W structure before humanizing. The black arrow indicates the end of the structural model obtained. B) The human structure of domains EC1-EC2 (PDB 2O72, blue) was aligned with the same domains of the human model generated from the xenopus structure (PDB 1L3W, red). Image created with Pymol. C) Schematic representation of human E-cadherin domains, highlighting the coverage of the three different structural models obtained with FoldX (models of prodomain, extracellular domain and the Catenin Binding Domain).

There are few human E-cadherin (hE-cad) structures available, and they only cover small portions of the protein (Table S1). Using automatic search of Swiss Model Repository, we found that PDB 1L3W, annotated for the full length extracellular domain of xenopus EP-cadherin (EP-cad), is highly homologous to the same domain in human E-cadherin. We analysed sequence homology by alignment using M-coffee, a multiple sequence alignment that combines the output of several multiple sequence alignment packages (PCMA, Poa, Mafft, Muscle, T-Coffee, ClustalW, ProbCons, DialignTX) [23], [24]. Figure 1A shows the alignment of the two extracellular domain sequences. The red brick regions represent perfect agreement among the methods used, representing highly similar sequences. To build the model structure, we removed regions with no similarity (Figure 1A, stars), and limited the model to regions with reliable alignment (black arrow, Figure 1A). The xenopus structure was humanized as described in Material and Methods and Figure 1B shows the structural alignment of hE-cad EC1–EC2 domains (from PDB 2O72) and EC1–EC2 domains of the xenopus derived structural model. The two structures are nearly superimposed, indicating that the similarity between the extracellular domains of human E-cadherin and xenopus EP-cadherin is not only at the sequence level but also at the structural level. The model structure of human E-cad exhibits compatible energies with the structure from xenopus, with a slight decrease of free energy (ΔG) obtained for the model (ΔG_real_ = 559.99 kcal/mol and ΔG_model_ = 531.77 kcal/mol), indicating that the humanization doesn't introduce extra clashes. Recently, a structure of the mouse extracellular domain was released (PDB 3Q2V, Table S1), and we also used this structure as a model, as a way to refine the results obtained with the xenopus model.

We established two other models, covering the Prodomain (PDB 1OP4, from mouse N-cadherin) and the β-catenin cytoplasmic domain (PDB 1I7X, from mouse E-cadherin), using the same methodology. Altogether, the three models cover most of the protein structure (Figure 1C): the prodomain model covers positions 28–117, the extracellular models positions 155–697 and the β-catenin cytoplasmic domain covers 782–838. At the level of the juxtamembrane domain, one structure is annotated, comprising the interacting surface between E-cadherin and p120 [28]. This structure contains a small, 18 aminoacids long peptide (covering positions 756–773 on hE-cad), with very low structural content, factors that decrease the reliability of the energy calculations, so we discarded this structure from the analysis.

### 2. *In silico* prediction of the impact of cancer-associated E-cadherin USVs

E-cadherin mutations are not only the genetic cause of HDGC, but they are also frequently found in different types of sporadic cancers. We analyzed *in silico* the impact of all cancer-associated E-cadherin missense mutations that localize to the regions covered by the structural models generated: 22 germline mutations found in the settings of HDGC and EODGC, and 57 found in sporadic cancers. Germline E-cadherin USVs were collected from the literature, and some are personal communications of our lab. Some HDGC/EODGC mutations are not possible to model, due to the lack of structural information (e.g. the ones localized in the juxtamembrane domain of E-cadherin), and were not included in this analysis. Somatic mutations were collected from the Cancer Genome Project database, and contain mutations found in gastric and lobular breast cancer (the only two types of cancer associated to HDGC), but also other types of cancer such as synovial sarcoma or bile duct carcinoma (Table S2).

**Figure 2:**
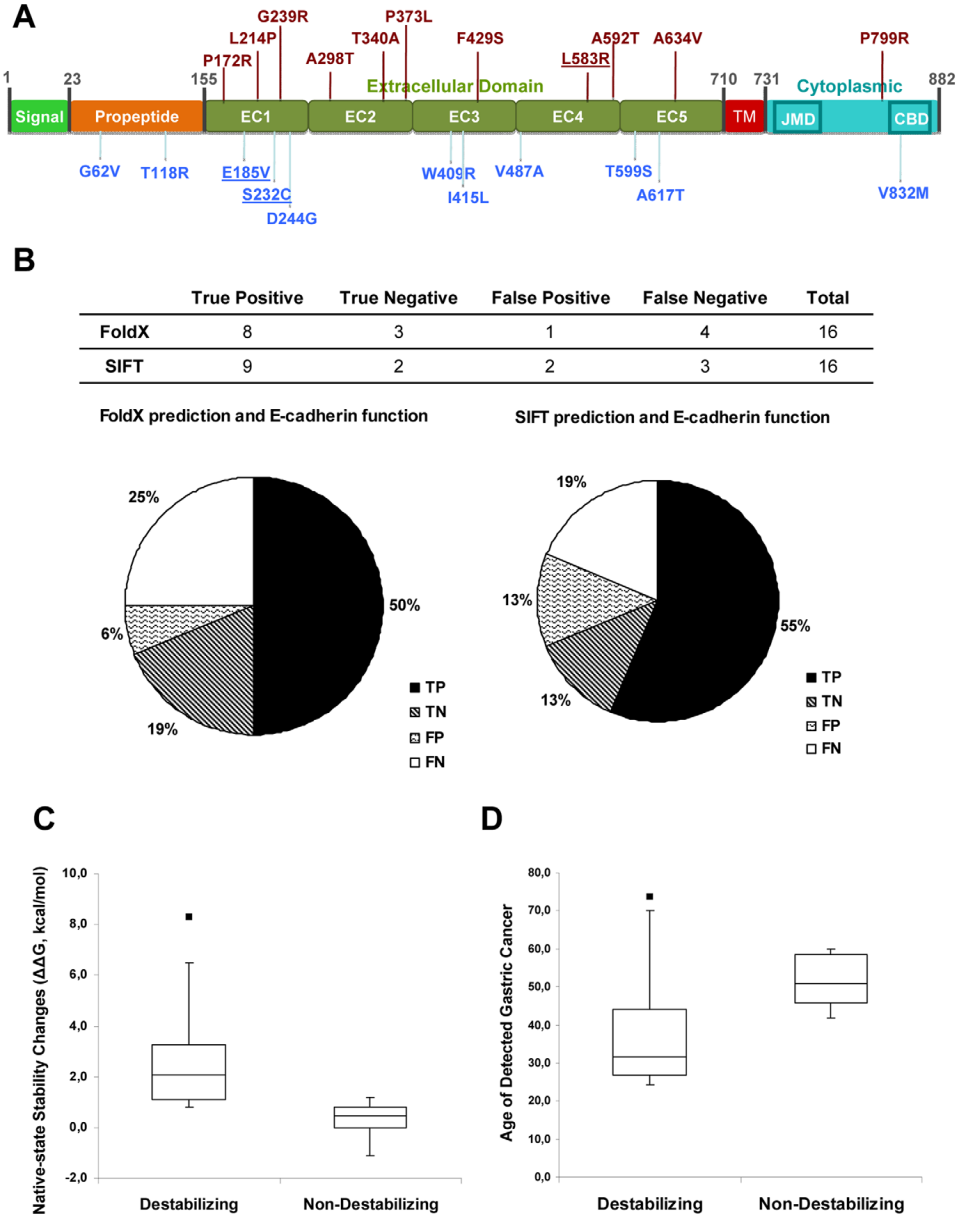
*In silico* analysis of the impact of germline E-cadherin missense mutations. A) Schematic representation of E-cadherin domains, mapping all the modelled germline mutations found in the setting of HDGC or EODGC. Above the scheme are the mutations that resulted in destabilization, as predicted by FoldX (ΔΔG>0.8 kcal/mol) and below the scheme all the non-destabilizing mutations (ΔΔG<0.8 kcal/mol). The newly identified mutations are underlined. B) FoldX and SIFT were used to evaluate the impact of the mutations present in A) and the predictions were classified as: True Positive (TP) when the software predicts high impact and the mutants exhibit in vitro loss of function; True Negative (TN) when the software predicts no impact and the mutant is functional in vitro; False Positive (FP) when the software predicts high impact but the mutants is functional in vitro; and False Negative (FN) when the software predicts no impact and the mutants exhibits in vitro loss of function. The results from both predictors result in 70% overlap with E-cadherin protein function tested in vitro (TP+TN). C) Box-plot representing the median and interquartile ranges of the native-state stability changes (ΔΔG) of the Destabilizing and Non-destabilizing mutations, as predicted by FoldX. D) Box-plot representing the median and interquartile ranges of ages of Gastric Cancer detection or associated death, corresponding to the Destabilizing and Non-destabilizing mutations carriers. All the data was collected from the literature. The group of patients harbouring destabilizing mutations is characterized by a clear younger age of diagnosis or death, suggesting the contribution of E-cadherin destabilization for the disease phenotype.

Using the structural models described previously, we used FoldX to generate each of the cancer associated USVs, and evaluated their native-state stability, ΔG (commonly referred as total energy for simplicity) [20]. The energetic difference between the WT reference and the corresponding mutant (ΔΔG = ΔG_WT_−ΔG_Mut_) was calculated for the 22 HDGC/EODGC E-cadherin USVs localized to the regions covered by the model structures, and the results are listed in Table 1. When ΔΔG is negative, this reflects a gain of native-state stability in the mutant form; when it is positive, it implies that the mutant is less stable then the WT reference. Previous studies in other proteins have shown that stability changes calculated with FoldX algorithm below 0.8 kcal/mol are within the error change of the software, and are thus considered to be non-significant [21]. Accordingly, we only considered mutations to be destabilizing when they induce energy changes above 0.8 kcal/mol. In Figure 2A, mutations above the scheme are destabilizing, while the bottom ones are structurally tolerated. It is clear that destabilizing mutations are spead along the protein, with no preferential domain affected.

**Table 1:** *In silico* based analysis of the impact of HDGC-associated E-cadherin missense mutations.

Aminoacid Alteration	Genetic Alteration	ΔΔG	SIFT Score	In vitro Function	FoldX	SIFT	Reference
**G62V**	c.185G>T	−0,45	0,14	ND	-	-	[41]
**T118R**	c.353C>G	−0,32	0,45	Loss	FN	FN	[42]
**P172R**	c.515C>G	0.8	0	ND	-	-	[43]
**L214P**	c.641T>C	7	0	Loss	TP	TP	(c)
**G239R^a^**	c.715G>A	2,56	0	Loss	TP	TP	[42]
**D244G^a^**	c.731A>G	0,15	0,14	ND	-	-	[44]
**A298T**	c.892G>A	8,31	0,01	Loss	TP	TP	[13]
**T340A**	c.1018A>G	1,09	0,08	Loss	TP	FN	[45], [46]
**P373L**	c.1118C>T	1,12	0	Loss	TP	TP	[47]
**W409R**	c.1225T>C	−0,24	0	Loss	FN	TP	[13]
**I415L**	c.1243A>C	−0,9	0,01	ND	-	-	[48]
**P429S**	c.1285C>T	1,56	0,02	Loss	TP	TP	[49]
**V487A**	c.1460T>C	0,45	0,56	ND	-	-	[44]
**A592T^b^**	c.1774G>A	3,42	0	Functional	FP	FP	[50], [51]
**T599S**	c.1796C>G	0,22	0,2	ND	-	-	[45]
**A617T^b^**	c.1849G>A	0,06	0,43	Functional	TN	TN	[17]
**A634V^a^**	c.1901C>T	1,05	0,26	Loss	TP	FN	[17], [52]
**P799R**	c.2396C>G	0,62	0,03	Loss	FN	TP	[50]
**V832M**	c.2494G>A	−1,1	0	Loss	FN	TP	[16], [53]
**E185V**	c.554A>T	0,29	0,25	Functional	TN	TN	(d)
**S232C**	c.695C>G	−0,9	0,01	Functional	TN	FP	(d)
**L583R**	c.1748T>G	2,72	0	Loss	TP	TP	[22]

Only mutations that localize in the domains covered by the structural models are listed. FoldX calculations are reflected by the value of native-state stability changes (ΔΔG = ΔG_WT_−ΔG_Mut_), expressed in kcal/mol. Mutations associated to structural impact present ΔΔG>0,8 kcal/mol in the FoldX column, and values below 0,05 in the SIFT column are considered to be intolerant due to high conservation. Predictions were scored as: True Positive (TP) when the software predicts high impact and the mutants exhibits in vitro loss of function; True Negative (TN) when the software predicts no impact and the mutant is functional in vitro; False Positive (FP) when the software predicts high impact but the mutant is functional in vitro; and False Negative (FN) when the software predicts no impact and the mutant exhibits in vitro loss of function. Only mutations that have been functionally characterized in vitro are classified. The mutations that have been described to impact the splicing pattern are depicted with (a). Mutations found at a frequency higher than 1% in one control population are considered polymorphisms, and marked with (b). Mutations published as personal communications are referenced with (c). The newly identified mutations are listed in the bottom of the table, unpublished are marked with a (d). ND – Not Determined.

The prodomain of hE-cad is cleaved during maturation, and if this is not accomplished, E-cadherin adhesive function is impaired [29], [30]. For the mutations localized in this domain, we evaluated the impact in total energy with FoldX, the conservation with SIFT, and also evaluated if the interference with the prodomain cleavage site with ProP (details in Materials and Methods). We found that both hereditary (G62V and T118R) and sporadic (P30T, G62D, H92Y, H121R and H123Y) mutations localized in E-cadherin prodomain are structurally tolerated, as predicted by FoldX (Table S3). When we analyse the impact based on conservation using SIFT, we also found that none of the mutations was considered deleterious, because their degree of conservation is low (Table S3). These results indicate that the pathogenicity of E-cadherin USVs localized in the prodomain is likely not dependent on destabilization. We also found no effect on the cleavage of the propeptide, as predicted by ProP (data not shown). Accordingly, we believe that the pathogenicity of E-cadherin mutations in this domain can result from the interference with the docking of proteins involved in prodomain processing, impossible to predict *in silico*.

Hereditary E-cadherin USVs span the full length of the extracellular domain, while sporadic mutations are predominantly found in EC2–EC3, as in accordance with the hotspot previously described in exons 7–9 [5]. From the total 18 germline HDGC/EODGC mutations localized in this domain, we found that 10 have a significant structural impact in the protein (Table 1). Approximately half of the sporadic mutations are also destabilizing (Table S3), independently of the EC domain where they are localized, suggesting that native-state destabilization may be associated to a substantial fraction of sporadic cancers involving E-cadherin loss by point mutation.

Only three mutations are localized in the region mapped by the model of the cytoplasmic β-catenin binding domain (P799R, V832M, S838G): the first two identified in the HDGC/EODGC setting and the other one sporadic, found in ovary carcinoma [31]. For these mutations we analysed the binding energy between E-cadherin and β-catenin and found that none of them significantly alters the binding affinity of β-catenin, according to FoldX prediction. This is in accordance with the *in vitro* results showing that the hereditary mutation V832M efficiently binds β-catenin, and its pathogenicity seems to be dependent on the inability of the E-cadherin/β-catenin complex to bind α-catenin [32], [33].

We collected all the predictions and functional *in vitro* data of HDGC/EODGC mutations and analysed the reliability of the predictors used (Table 1). We classified the results from the predictions as: True Positive (TP) when the mutation is predicted as deleterious *in silico* (either by FoldX or SIFT) and exhibit loss of function *in vitro*; True Negative (TN) when the mutation is predicted as tolerated *in silico* and is functional *in vitro*; False Positive (FP) when the mutation is predicted as deleterious *in silico* but is functional *in vitro*; and False Negative (FN) when the mutation is predicted as tolerated *in silico* but exhibits *in vitro* loss of function. TP and TN are positive results, meaning that the predictors are able to detect the mutation impact in function; FP and FN represent their degree of failure. We found that both algorithms are able to predict the functional impact of up to 70% of the germline HDGC/EODGC mutations (11 out of 16 mutations), with predictions overlapping for half of the mutations (Table 1, Figure 2B).

We analysed the data available for the germline HDGC/EODGC mutations carriers and, although the information is limited, we found that the most complete set of data is the age of onset or death associated to DGC. When we box-plot this data, grouping “Destabilizing” and “Non-destabilizing” mutation carriers, we observe an evident younger age of disease onset (diagnosis or death) for the first group (Figure 2D), suggesting that native-state destabilization accounts for the earlier development of DGC.

### 3. Biological significance of E-cadherin destabilization

To determine the biological significance of E-cadherin destabilization, we used as model system three newly identified E-cadherin germline missense mutations reported to our lab for functional characterization: E185V, S232C and L583R, the later recently described in the literature [22]. The *in silico* analysis described previously was performed for these three new mutations and the results are included in Table 1 (below the dark line). Mutations E185V and S232C are structurally tolerated, with ΔΔG = 0.29 kcal/mol and −0.9 kcal/mol respectively (Table 1), considered insignificant regarding the impact in structure. Mutation S232C promotes a decrease in energy, stabilizing the protein, and this is due to the loss of the high energy of a Serine buried OH group, which is not involved in an H-bond, and to the accommodation of the sidechain of Cysteine. Mutation L583R induces destabilization, with ΔΔG = 2.72 kcal/mol, reflecting the dramatic change from a hydrophobic to hydrophilic aminoacid, that results in an Arginine not able to form H-bonds, being unfavourably buried.

**Figure 3:**
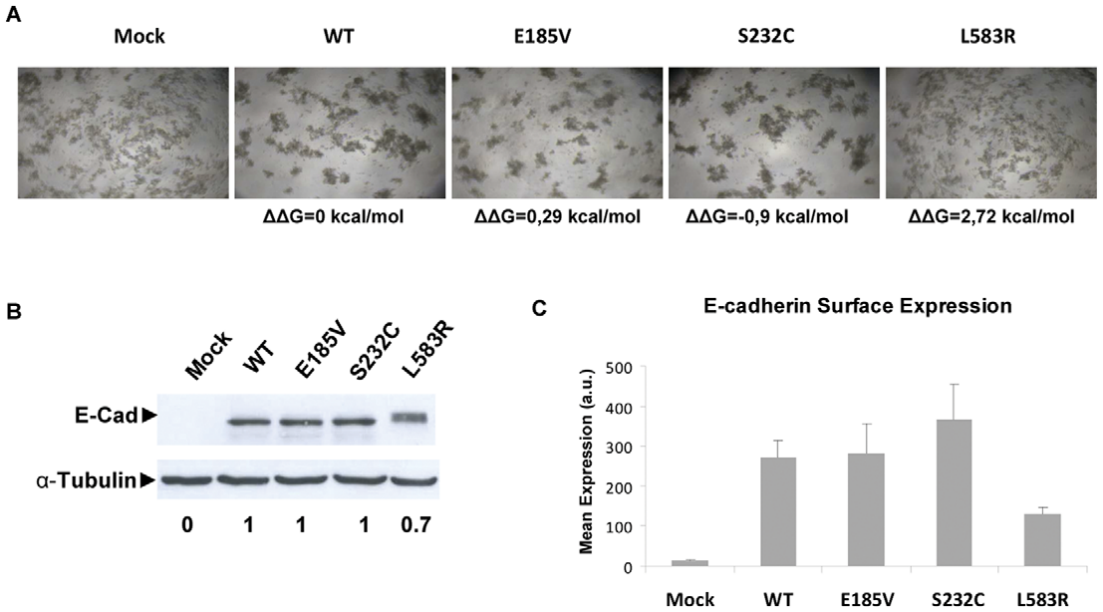
Functional impact of three new HDGC-associated E-cadherin missense mutations: E185V, S232C and L583R. CHO cells were transiently (A) or stably (B–C) transfected with an empty vector (Mock) or WT, E185V, S232C, L583R E-cadherin cDNA. A) Functional aggregation assay was performed as described in Material and Methods. L583R cells show E-cadherin loss of function, resulting in a scattered pattern, resembling Mock cells. ΔΔG was calculated using FoldX algorithm and is 0 for the WT reference; B) Total cell lysates were prepared and E-cadherin was detected by Western Blot using anti-E-cadherin antibody. Anti-α-Tubulin antibody was used as a loading control. The expression of L583R is reduced and shifted to higher molecular weight, indicative of being retained as immature (approximately 130 kDa). C) E-cadherin expression in the Plasma Membrane (PM) was evaluated using Flow Cytometry, after staining with an extracellular anti-human E-cadherin antibody. L583R is less expressed in the PM.

I*n vitro* functional assays were performed for the above-mentioned HDGC/EODGC mutations, and we found a perfect correlation between the functionality *in vitro* and the presence/absence of structural impact: E185V and S232C retain the adhesive function of E-cadherin, and are able to form tight cellular aggregates, while L583R exhibits a clear scattered pattern, resembling Mock cells (Figure 3A), indicating that E185V and S232C are non-pathogenic and L583R is pathogenic.

When we analysed E-cadherin expression in the different cell lines, we found that the total amount of mutant L583R is lower that the WT expression under the same conditions, while mutations E185V and S232C, retain normal levels (Figure 3B). Interestingly, the band corresponding to L583R is retained in the gel, indicating that L583R is not able to properly mature (immature form of E-cadherin is 130 kDa, mature is 120 kDa) and flow cytometry results show that it is less expressed in the plasma membrane (Figure 3C).

**Figure 4:**
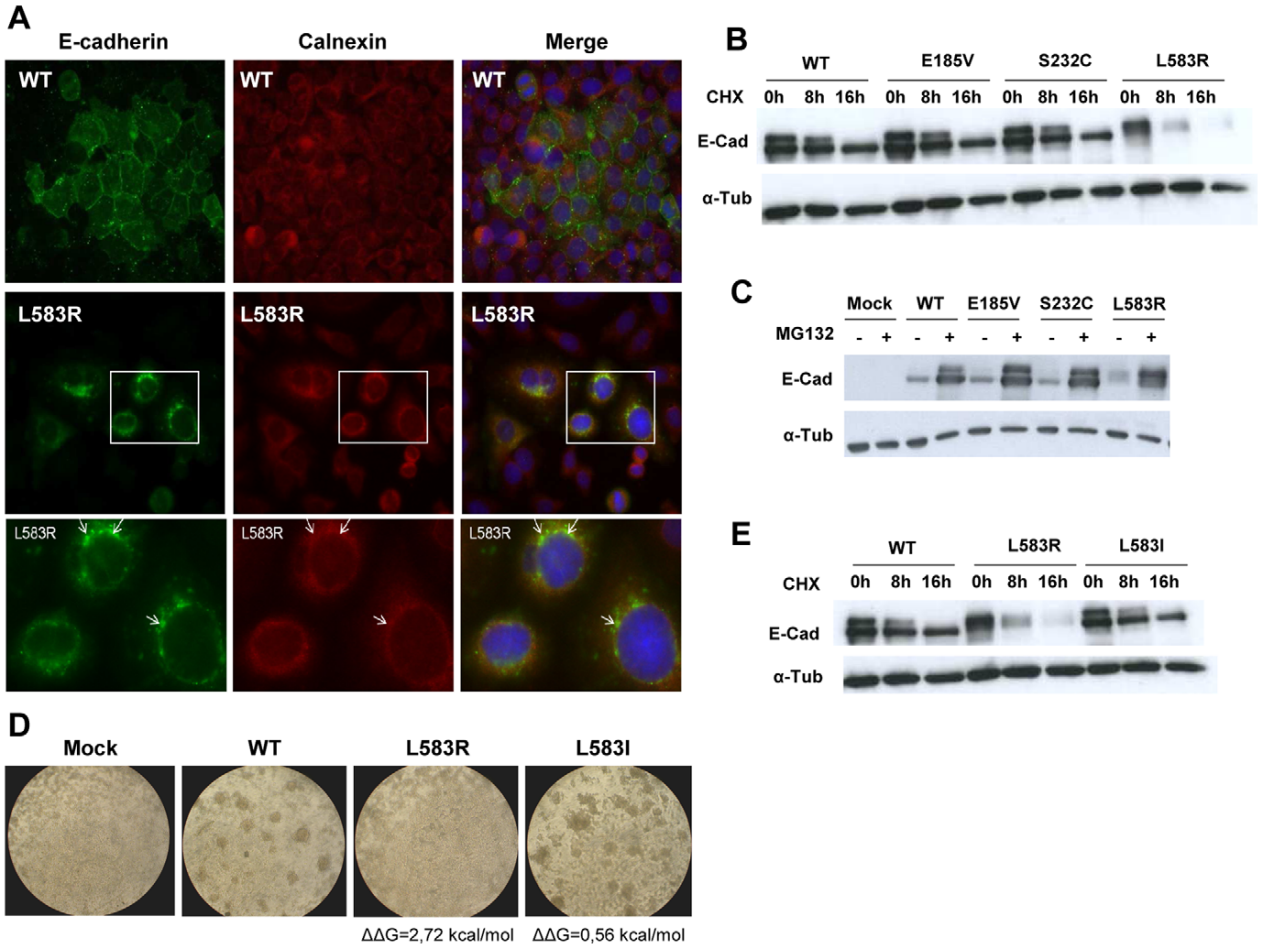
ERAD is involved in the regulation of E-cadherin destabilizing mutations. CHO cells were stably (A, C) or transiently (B, D, E) transfected with an empty vector (Mock) or WT, E185V, S232C, L583R, L583I E-cadherin cDNA. A) E-cadherin and Calnexin immunofluoresce was performed in stable CHO cells expressing WT and L583R. Calnexin was used as an ER marker. L583R is retained in the ER, as evaluated by the colocalization with calnexin (yellow and arrows). B) Protein synthesis was blocked with Cicloheximide for 8 h and 16 h, to analyse E-cadherin turnover. E-cadherin was detected by Western Blot using anti-E-cadherin antibody and anti-α-Tubulin antibody was used as a loading control. L583R exhibits higher turnover. C) Cells were incubated with proteasome inhibitor MG132 for 16 h, and total cell lysates were prepared and analyzed. Proteasomal degradation results on the accumulation of L583R to levels similar to WT, indicating that the proteasome is necessary for the mutant downregulation. D) Functional aggregation assay was performed as described in Material and Methods. Cells expressing the artificial mutant L583I recover E-cadherin adhesive function, resembling WT cells, in contrast to L583R, which are not able to perform adhesion. E) Protein synthesis was blocked with Cicloheximide for 8 h and 16 h, to analyse E-cadherin turnover. In contrast to the unstable L583R, the stable mutation (L583I) is resistant to protein synthesis blockage, exhibiting lower turnover, comparable to the WT protein. The two bands of E-cad in B) and E) correspond to mature (lower, 120 kDa) and immature (upper, 130 kDa) forms of the protein, and result from the overload of protein commonly observed upon transient transfections.

When protein maturation fails, this commonly results in Endoplasmic Reticulum (ER) retention of immature protein. To test whether L583R was indeed retained as immature, we analysed if it is retained in the ER by co-immunofluorescence with the ER marker calnexin (Figure 4A), and found that part of the L583R signal is superimposed with the ER marker, indicating increased ER retention.

To understand if destabilization could be detected *in vitro*, we analysed the stability of L583R in the cell, evaluating its turnover. We blocked protein synthesis with cycloheximide and found that L583R is soon degraded, as evaluated by its residual expression soon after 8 h of protein synthesis inhibition, in contrast to WT and the other mutants that are still highly expressed in the same condition (Figure 4B), indicating that L583R is unstable in the cell. The presence of immature band in WT or mutant E-cadherin samples (top band, Figure 4B) is due to the overload of protein resulting from transient transfection.

Unstable or misfolded proteins are tightly regulated by Protein Quality Control mechanisms that protect the cell by directing newly synthesized unfolded proteins for degradation in the proteasome [34]. To address if this is the case for L583R, we inhibited the proteasome activity with MG132 and observed that, despite the different initial levels of E-cadherin, the expression of mutant L583R is completely restored upon treatment (Figure 4C), indicating that it is prematurely degraded by the proteasome after synthesis, as previously described for other juxtamembranar HDGC-associated E-cadherin mutations [35]. Interestingly, when proteasome degradation is inhibited, there is an accumulation of immature E-cad in all cell lines, manifesting the importance of the proteasome in the regulation of newly synthesized E-cad, independently of being mutated or not.

To further validate the *in silico* predictions, we analysed the phenotype of a reverted destabilized mutation by inducing a structurally tolerated alteration in the same position of the mutant form of E-cadherin. Using FoldX, we calculated the impact of each possible alteration in position 583 and found that the alteration inducing less destabilization was L583I (ΔΔG = 0.56 kcal/mol, as predicted using the mouse model, Table S3). Interestingly, this mutation retains the adhesive function of E-cadherin, resulting in compact cell aggregates (Figure 4D), and is not destabilized *in vitro*, exhibiting cicloheximide resistance comparable to the WT form (Figure 4E). These results emphasize the reliability of the *in silico* based predictions of E-cadherin stability and the clear association of E-cadherin destabilization with loss of adhesive function.

## Discussion

E-cadherin alterations (mutations, deletions and methylation) are the only recognized genetic cause of HDGC [36], [37], [38]. Most mutations identified in HDGC are of the nonsense type, but a significant proportion (20%) of germline mutations give rise to single aminoacid substitutions, of which the pathogenicity is difficult to evaluate and is often unclear [39], [40]. The most important information in terms of genetic counselling of germline missense mutation carriers is familial clinical information (segregation analysis, mostly) but this information is often scarce with the size of the pedigree commonly being too small to allow segregation studies, and the pathogenicity assessment usually comes from cell-based *in vitro* functional assays [15], [17], which are time consuming and technically demanding, and are therefore not widely applicable in routine molecular labs. Consequently, there is a need for new methods to determine the pathogenicity of E-cadherin missense mutations associated to HDGC [40]. Our group has previously described a model to infer the pathogenicity of this type of mutation, based on different variants such as co-segregation of the mutation within pedigrees, frequency in healthy population, recurrence in independent families, and functional in vitro and in silico data [15]. In that case, the structural in silico analysis was limited to the EC1–EC2 domains, and was thus incomplete. In this work we used FoldX to generate structural models covering the major part of E-cadherin (Figure 1C), calculate the energetic penalty induced by each mutation, and compare the results with in vitro and in vivo phenotypes.

We have previously showed that E-cadherin folding is surveyed by mechanisms of Protein Quality Control and that HDGC-associated mutations can be prematurely degraded by the Endoplasmic Reticulum Associated Degradation (ERAD), a mechanism responsible for the clearance of misfolded and unstable proteins, dependent on the proteasome [35]. These results strongly suggest that some E-cadherin missense mutations may have structural impact, resulting in protein misfolding. To evaluate if each mutation impacts the structure and stability of E-cadherin, we based our analysis in the calculation of native-state stability changes (ΔΔG) using FoldX algorithm. We evaluated 22 germline HDGC/EODGC and 57 sporadic E-cadherin missense mutations regarding structural impact (FoldX) and evolutionary conservation (based on SIFT analysis) and found that the destabilizing mutations span the full length of the extracellular domain, with no hotspot for a particular domain. Most HDGC/EODGC mutations (16 out of 22) are functionally characterized *in vitro*, so we used this information to evaluate the power of the predictors to infer loss of function. Using a simple classification of the results obtained with FoldX and SIFT (True Positive, True Negative, False Positive and False Negative), we found that both predictors accurately predict around 70% of the functional impact of the mutations (TP plus TN); with FoldX, we found that loss of function correlates with loss of native-state stability for half of the analysed mutations (8 out of 16), with most of them also being highly conserved (6 out of 8). Interestingly, when we analysed the mutation carriers in detail, we found that the group of patients harbouring Destabilizing mutations is characterized by the development of disease at a younger age, when compared to the group with mutations that keep native-state stability (Figure 2D). These results indicate that not only E-cadherin native-state stability is frequently disturbed by germline mutations, but also that destabilization accounts for the disease phenotype, inducing earlier development of disease. It would be informative if imunohistochemistry of E-cadherin in DGC was compared between carriers of “Destabilizing” and “Non-destabilizing” mutations, but we didn't have enough material to analyze this parameter in depth. Additionally, the percentage of destabilizing sporadic mutations is also high (around 40%), suggesting that destabilization may also account for the loss of E-cadherin function in sporadic cancer.

Furthermore, we wanted to analyse if E-cadherin structural destabilization induced by missense mutations was correlated to misfolding in the cell and recognition by ERAD, resulting in loss of expression. We used three newly identified germline mutants (E185V, S232C and L583R) as model, and evaluated their impact *in silico* with FoldX and SIFT, and *in vitro*, characterizing expression, subcellular localization and degradation pattern. We found that only mutation L583R induces high impact in the structure (ΔΔG = 2,7 kcal/mol) besides being totally conserved. *In vitro* functional assays indicate that L583R is pathogenic, exhibiting loss of adhesive function (scattered cell pattern), in contrast to E185V and S232C that retain adhesive function (Figure 3). We found that L583R is less expressed due to maturation deficiency, accumulates in the ER, is prematurely degraded by the proteasome, and exhibits high turnover and shorter half-life, indicating that it is regulated by ERAD (Figure 4). Interestingly, if we introduce a structurally tolerated alteration in the same position (L583I, as predicted by FoldX), the function is recovered and stability is restored, resulting in an increased in half-life of the protein (Figure 4D–E). This result shows the direct correlation between *in silico* predicted destabilization and decreased E-cadherin half-life.

Overall, our results indicate that E-cadherin missense mutations found in cancer frequently lead to native-state destabilization, and we show that the carriers of destabilizing mutations develop DGC earlier in life, suggesting that this subset of mutations is more pathogenic. *In vitro* studies show that structural destabilization results in high turnover in the cell, recognition by ERAD, premature degradation by the ubiquitin-proteasome system and consequent loss-of-function. We propose for the first time that E-cadherin destabilization accounts for HDGC pathogenicity, and that in the absence of clear clinical observations, *in silico* predictions should be used as a first approach to distinguish pathogenic from probably tolerated E-cadherin variants associated to HDGC or EODGC.

## Supporting Information

Table S1 **E-Cadherin related Protein Data Bank structures available.**
(DOC)

Table S2 **E-cadherin missense mutations in sporadic cancers.** This table lists all the E-cadherin missense mutations localized in the domains covered by the structural models generated in this study. They were collected from Cosmic, a database for somatic mutations, as part of the Cancer Genome Project (details in Material and Methods). Pro, prodomain; EC, Cadherin domain; CBD, Catenin Binding Domain; ND, Not Determined
(DOC)

Table S3 **In silico predictions for all cancer-associated E-cadherin USVs by FoldX (ΔΔG = ΔG_WT_−ΔG_Mut_) and SIFT.** Structural impact is considered when ΔΔG>0,8 kcal/mol in the FoldX column, and values bellow 0,05 in the SIFT column are considered to be intolerant due to high conservation. Newly identified HDGC-associated mutations are listed on the bottom of the table, with unpublished marked with (a). **ΔΔG_model_** is the stability change as calculated in the models described in Material and Methods; **ΔΔG_mouse_** contains the results when the calculations are made in Chain B of a recent PDB annotated for mouse E-cadherin extracellular domain (3Q2V), using a similar method. Italic numbers were calculated in Chain A, due to lack of coverage in Chain B. HDGC, Hereditary Diffuse Gastric Cancer; CBD – Cadherin Binding Domain.
(DOC)
