# Supplementary material for: E-Cadherin Destabilization Accounts for the Pathogenicity of Missense Mutations in Hereditary Diffuse Gastric Cancer
Source: PLoS One. 2012 Mar 21;7(3):e33783. doi: 10.1371/journal.pone.0033783 (PMC3309996; doi:10.1371/journal.pone.0033783)
Supplement: Table S1 — E-Cadherin related Protein Data Bank structures available. (DOC) [file pone.0033783.s001.doc]

**Supporting Table 1. E-Cadherin related Protein Data Bank structures available**

| **PDB Entry** | **Protein** | **Method** | **Resolution (Å)** | **Chain** | **Positions** | **Domains** | **Origin** |
| --- | --- | --- | --- | --- | --- | --- | --- |
| 1O6S | E-cadherin | X-ray | 1.80 | B | 156-255 | EC1 | Human |
| 2O72 | E-cadherin | X-ray | 2.00 | A | 155-367 | EC1-EC2 | Human |
| 2OMT | E-cadherin | X-ray | 2.00 | B | 156-255 | EC1 | Human |
| 2OMU | E-cadherin | X-ray | 1.80 | B | 156-255 | EC1 | Human |
| 2OMV | E-cadherin | X-ray | 1.90 | B | 156-255 | EC1 | Human |
| 2OMX | E-cadherin | X-ray | 1.70 | B | 156-258 | EC1 | Human |
| 2OMY | E-cadherin | X-ray | 1.70 | B | 156-255 | EC1 | Human |
| 2OMZ | E-cadherin | X-ray | 1.60 | B | 156-255 | EC1 | Human |
| 3FF7 | E-cadherin | X-ray | 1.80 | A/B | 155-253 | EC1 | Human |
| 3FF8 | E-cadherin | X-ray | 2.00 | A/B | 155-254 | EC1 | Human |
| 3L6X | E-cadherin | X-ray | 2.40 | B | 756-773 | Catenin Binding | Synthetic |
| 3L6Y | E-cadherin | X-ray | 3.00 | B/D/F | 756-773 | Catenin Binding | Synthetic |
| 1EDH | E-cadherin | X-ray | 2.00 | A/B | 156-380 | EC1-EC2 | Mouse |
| 1FF5 | E-cadherin | X-ray | 2.93 | A/B | 157-374 | EC1-EC2 | Mouse |
| 1I7W | E-cadherin | X-ray | 2.00 | B/D | 734-884 | Catenin Binding | Mouse |
| 1I7X | E-cadherin | X-ray | 3.00 | B/D | 734-884 | Catenin Binding | Mouse |
| 1Q1P | E-cadherin | X-ray | 3.20 | A | 158-369 | EC1-EC2 | Mouse |
| 1SUH | E-cadherin | NMR | - | A | 156-300 | EC1-EC2 | Mouse |
| 2OMW | E-cadherin | X-ray | 1.85 | B | 158-256 | EC1 | Mouse |
| 2QVF | E-cadherin | X-ray | 2.40 | B | 157-369 | EC1-EC2 | Mouse |
| 3IFQ | E-cadherin | X-ray | 2.80 | C/D | 778-884 | Catenin Binding | Mouse |
| 3LNE | E-cadherin | X-ray | 2.00 | A | 157-369 | EC1-EC2 | Mouse |
| 3LNF | E-cadherin | X-ray | 2.50 | A/B | 159-369 | EC1-EC2 | Mouse |
| 3LNG | E-cadherin | X-ray | 2.70 | A/B | 157-369 | EC1-EC2 | Mouse |
| 3LNH | E-cadherin | X-ray | 2.60 | A/B | 159-369 | EC1-EC2 | Mouse |
| 3LNI | E-cadherin | X-ray | 2.30 | A/B | 157-369 | EC1-EC2 | Mouse |
| 1L3W | EP-cadherin | X-ray | 3.08 | A | 156-695 | EC1-EC5 | Xenopus |
| 1OP4 | N-cadherin | NMR | - | A | 24-159 | Prodomain | Mouse |
| 3Q2V | E-cadherin | X-ray | 3,40 | A/B | 155-698 | EC1-EC5 | Mouse |
