# Supplementary material for: E-Cadherin Destabilization Accounts for the Pathogenicity of Missense Mutations in Hereditary Diffuse Gastric Cancer
Source: PLoS One. 2012 Mar 21;7(3):e33783. doi: 10.1371/journal.pone.0033783 (PMC3309996; doi:10.1371/journal.pone.0033783)
Supplement: Table S2 — E-cadherin missense mutations in sporadic cancers. This table lists all the E-cadherin missense mutations localized in the domains covered by the structural models generated in this study. They were collected from Cosmic, a database for somatic mutations, as part of the Cancer Genome Project (details in Material and Methods). Pro, prodomain; EC, Cadherin domain; CBD, Catenin Binding Domain; ND, Not Determined (DOC) [file pone.0033783.s002.doc]

**Supporting Table 2. E-cadherin missense mutations in sporadic cancers**

| **Domain** | **Mutation** | **Tissue** | **Histology** | **References** |
| --- | --- | --- | --- | --- |
| **Pro** | P30T | Breast | Lobular Carcinoma | [1] |
| **Pro** | G62D | Breast | Lobular Carcinoma in situ | [2] |
| **Pro** | H92Y | Breast | Lobular Carcinoma in situ | [2] |
| **Pro** | H121R | Breast | Lobular Carcinoma in situ | [2] |
| **Pro** | H123Y | Stomach | Diffuse Adenocarcinoma | [3] |
| **EC1** | P159S | Soft tissue | Synovial Sarcoma | [4] |
| **EC1** | P170L | Soft tissue | Synovial Sarcoma | [4] |
| **EC1** | T193P | Stomach | Signet Ring Adenocarcinoma | [5] |
| **EC1** | P245L | Breast | Lobular Carcinoma in situ | [6] |
| **EC1** | D254Y | Breast | Lobular Carcinoma in situ | [2] |
| **EC1** | Q255H | Soft tissue | Synovial Sarcoma | [4] |
| **EC1** | D257G | Breast | Lobular Carcinoma in situ | [6] |
| **EC1** | P260L | Breast | Ductal Carcinoma in situ | [6] |
| **EC2** | S270A | Prostate | ND | [7] |
| **EC2** | M282I | Breast | Ductal Carcinoma in situ | [8] |
| **EC2** | A286T | Breast | Lobular Carcinoma in situ | [2,6] |
| **EC2** | T295I | Biliary Tract | Bile Duct Adenocarcinoma | [9] |
| **EC2** | A298D | Breast | Lobular Carcinoma in situ | [6] |
| **EC2** | I300M | Biliary Tract | Bile Duct Adenocarcinoma | [9] |
| **EC2** | D308N | Breast | Lobular Carcinoma in situ | [6] |
| **EC2** | P309S | Biliary Tract | Bile Duct Adenocarcinoma | [9] |
| **EC2** | L311F | Soft tissue | Synovial Sarcoma | [4] |
| **EC2** | N315S | Breast | Lobular Carcinoma | [10] |
| **EC2** | T330I | Breast | Lobular Carcinoma in situ | [2] |
| **EC2** | D334H | Stomach | Diffuse Adenocarcinoma | [11] |
| **EC2** | P339L | Soft tissue | Synovial Sarcoma | [4] |
| **EC2** | T340M | Biliary Tract | Bile Duct Adenocarcinoma | [9] |
| **EC2** | T340A | Soft tissue | Alveolar Soft Part Sarcoma | [12] |
| **EC2** | V344M | Soft tissue | Synovial Sarcoma | [4] |
| **EC2** | A347V | Stomach | Diffuse Adenocarcinoma | [11] |
| **EC2** | E353K | Soft tissue | Synovial Sarcoma | [4] |
| **EC2** | T357I | Biliary Tract | Bile Duct Adenocarcinoma | [9] |
| **EC2** | V365L | Stomach | Mixed Adenocarcinoma | [11] |
| **EC2** | N369D | Stomach | Mixed Adenocarcinoma | [11] |
| **EC2** | D370H | Stomach | Mixed/Diffuse Adenocarcinoma | [11] |
| **EC3** | D370A | Stomach | Diffuse Adenocarcinoma | [13] |
| **EC3** | P385L | Soft tissue | Synovial Sarcoma | [4] |
| **EC3** | E386K | Soft tissue | Synovial Sarcoma | [4] |
| **EC3** | T399I | Soft tissue | Synovial Sarcoma | [4] |
| **EC3** | D400Y | Stomach | Mixed/Diffuse Adenocarcinoma | [11] |
| **EC3** | A401T | Soft tissue | Synovial Sarcoma | [4] |
| **EC3** | D402N | Stomach | Diffuse Adenocarcinoma | [11] |
| **EC3** | T414I | Soft tissue | Synovial Sarcoma | [4] |
| **EC3** | V456M | Breast | Lobular Carcinoma in situ | [2] |
| **EC3** | E463Q | Stomach | Diffuse Adenocarcinoma | [3] |
| **EC3** | V473D | Stomach | Diffuse Adenocarcinoma | [13] |
| **EC4** | D479G | Stomach | Diffuse Adenocarcinoma | [11] |
| **EC4** | S559N | Breast | Lobular Carcinoma in situ | [2] |
| **EC4** | G571S | Breast | Lobular Carcinoma | [14] |
| **EC4** | L581P | Stomach | Diffuse Adenocarcinoma | [11] |
| **EC4** | A592T | Stomach/Thyroid | Intestinal Adenocarcinoma and Papillary Carcinoma | [15,16] |
| **EC5** | R598Q | Stomach | Diffuse Adenocarcinoma | [3] |
| **EC5** | I600M | Breast | Lobular Carcinoma in situ | [2] |
| **EC5** | A617T | Endometrium | Endometrium Carcinoma | [17] |
| **EC5** | A634V | Colon/Stomach | Colon and Gastric Carcinoma Cell line | [18,19] |
| **EC5** | A692V | Breast | Lobular Carcinoma in situ | [2] |
| **CBD** | S838G | Ovary | Ovary Carcinoma | [17] |

This table lists all the E-cadherin missense mutations localized in the domains covered by the structural models generated in this study. They were collected from Cosmic, a database for somatic mutations, as part of the Cancer Genome Project (details in Material and Methods). Pro, prodomain; EC, Cadherin domain; CBD, Catenin Binding Domain; ND, Not Determined

1. Sarrio D, Moreno-Bueno G, Hardisson D, Sanchez-Estevez C, Guo M, et al. (2003) Epigenetic and genetic alterations of APC and CDH1 genes in lobular breast cancer: relationships with abnormal E-cadherin and catenin expression and microsatellite instability. Int J Cancer 106: 208-215.

2. Mastracci TL, Tjan S, Bane AL, O'Malley FP, Andrulis IL (2005) E-cadherin alterations in atypical lobular hyperplasia and lobular carcinoma in situ of the breast. Mod Pathol 18: 741-751.

3. Becker KF, Reich U, Schott C, Becker I, Berx G, et al. (1999) Identification of eleven novel tumor-associated E-cadherin mutations. Mutations in brief no. 215. Online. Hum Mutat 13: 171.

4. Saito T, Oda Y, Sugimachi K, Kawaguchi K, Tamiya S, et al. (2001) E-cadherin gene mutations frequently occur in synovial sarcoma as a determinant of histological features. Am J Pathol 159: 2117-2124.

5. Muta H, Noguchi M, Kanai Y, Ochiai A, Nawata H, et al. (1996) E-cadherin gene mutations in signet ring cell carcinoma of the stomach. Jpn J Cancer Res 87: 843-848.

6. Rieger-Christ KM, Pezza JA, Dugan JM, Braasch JW, Hughes KS, et al. (2001) Disparate E-cadherin mutations in LCIS and associated invasive breast carcinomas. Mol Pathol 54: 91-97.

7. Ikonen T, Matikainen M, Mononen N, Hyytinen ER, Helin HJ, et al. (2001) Association of E-cadherin germ-line alterations with prostate cancer. Clin Cancer Res 7: 3465-3471.

8. Lei H, Sjoberg-Margolin S, Salahshor S, Werelius B, Jandakova E, et al. (2002) CDH1 mutations are present in both ductal and lobular breast cancer, but promoter allelic variants show no detectable breast cancer risk. Int J Cancer 98: 199-204.

9. Endo K, Ashida K, Miyake N, Terada T (2001) E-cadherin gene mutations in human intrahepatic cholangiocarcinoma. J Pathol 193: 310-317.

10. Kanai Y, Oda T, Tsuda H, Ochiai A, Hirohashi S (1994) Point mutation of the E-cadherin gene in invasive lobular carcinoma of the breast. Jpn J Cancer Res 85: 1035-1039.

11. Machado JC, Soares P, Carneiro F, Rocha A, Beck S, et al. (1999) E-cadherin gene mutations provide a genetic basis for the phenotypic divergence of mixed gastric carcinomas. Lab Invest 79: 459-465.

12. Saito T, Oda Y, Kawaguchi K, Takahira T, Yamamoto H, et al. (2003) Possible association between tumor-suppressor gene mutations and hMSH2/hMLH1 inactivation in alveolar soft part sarcoma. Hum Pathol 34: 841-849.

13. Becker KF, Atkinson MJ, Reich U, Becker I, Nekarda H, et al. (1994) E-cadherin gene mutations provide clues to diffuse type gastric carcinomas. Cancer Res 54: 3845-3852.

14. Droufakou S, Deshmane V, Roylance R, Hanby A, Tomlinson I, et al. (2001) Multiple ways of silencing E-cadherin gene expression in lobular carcinoma of the breast. Int J Cancer 92: 404-408.

15. Ascano JJ, Frierson H, Jr., Moskaluk CA, Harper JC, Roviello F, et al. (2001) Inactivation of the E-cadherin gene in sporadic diffuse-type gastric cancer. Mod Pathol 14: 942-949.

16. Soares P, Berx G, van Roy F, Sobrinho-Simoes M (1997) E-cadherin gene alterations are rare events in thyroid tumors. Int J Cancer 70: 32-38.

17. Risinger JI, Berchuck A, Kohler MF, Boyd J (1994) Mutations of the E-cadherin gene in human gynecologic cancers. Nat Genet 7: 98-102.

18. Vecsey-Semjen B, Becker KF, Sinski A, Blennow E, Vietor I, et al. (2002) Novel colon cancer cell lines leading to better understanding of the diversity of respective primary cancers. Oncogene 21: 4646-4662.

19. Milne AN, Sitarz R, Carvalho R, Polak MM, Ligtenberg M, et al. (2007) Molecular analysis of primary gastric cancer, corresponding xenografts, and 2 novel gastric carcinoma cell lines reveals novel alterations in gastric carcinogenesis. Hum Pathol 38: 903-913.
