# Supplementary material for: E-Cadherin Destabilization Accounts for the Pathogenicity of Missense Mutations in Hereditary Diffuse Gastric Cancer
Source: PLoS One. 2012 Mar 21;7(3):e33783. doi: 10.1371/journal.pone.0033783 (PMC3309996; doi:10.1371/journal.pone.0033783)
Supplement: Table S3 — In silico predictions for all cancer-associated E-cadherin USVs by FoldX (ΔΔG = ΔGWT−ΔGMut) and SIFT. Structural impact is considered when ΔΔG>0,8 kcal/mol in the FoldX column, and values bellow 0,05 in the SIFT column are considered to be intolerant due to high conservation. Newly identified HDGC-associated mutations are listed on the bottom of the table, with unpublished marked with (a). ΔΔGmodel is the stability change as calculated in the models described in Material and Methods; ΔΔGmouse contains the results when the calculations are made in Chain B of a recent PDB annotated for mouse E-cadherin extracellular domain (3Q2V), using a similar method. Italic numbers were calculated in Chain A, due to lack of coverage in Chain B. HDGC, Hereditary Diffuse Gastric Cancer; CBD – Cadherin Binding Domain. (DOC) [file pone.0033783.s003.doc]

**Supporting Table 3. In silico predictions of the impact of cancer associated E-cadherin USVs using FoldX (ΔΔG=ΔG_WT_- ΔG_Mut_) and SIFT**

| **Domain** | **Mutation** | **Genetic Alteration** | **Setting** | **ΔΔG_model_** | **ΔΔG_mouse_** | **SIFT** |
| --- | --- | --- | --- | --- | --- | --- |
| **Pro** | **P30T** | c.88C>A | Sporadic | 1,43 | - | 0,61 |
| **Pro** | **G62V** | c.185G>T | HDGC | -0,45 | - | 0,14 |
| **Pro** | **G62D** | c.185G>A | Sporadic | -0,37 | - | 0,11 |
| **Pro** | **H92Y** | c.274C>T | Sporadic | -0,23 | - | 0,53 |
| **Pro** | **T118R** | c.353C>G | HDGC | -0,32 | - | 0,45 |
| **Pro** | **H121R** | c.362A>G | Sporadic | -0,27 | - | 0,54 |
| **Pro** | **H123Y** | c.367C>T | Sporadic | -0,21 | - | 0,14 |
| **EC1** | **P159S** | c.475C>T | Sporadic | 1,08 | 1,2 | 0,01 |
| **EC1** | **P170L** | c.509C>T | Sporadic | 0,02 | -0,08 | 0 |
| **EC1** | **P172R** | c.515C>G | HDGC | 0,8 | 2,47 | 0 |
| **EC1** | **T193P** | c.577A>C | Sporadic | 3,83 | 2,13 | 0 |
| **EC1** | **L214P** | c.641T>C | HDGC | 7 | 8,21 | 0 |
| **EC1** | **G239R*** | c.715G>A | HDGC | 2,56 | 2,47 | 0 |
| **EC1** | **D244G*** | c.731A>G | HDGC | 0,15 | 0,24 | 0,14 |
| **EC1** | **P245L** | c.734C>T | Sporadic | 1,09 | 1,24 | 0 |
| **EC1** | **D254Y** | c.760G>T | Sporadic | 1,05 | -0,56 | 0 |
| **EC1** | **Q255H** | c.765G>T | Sporadic | 0,16 | -0,44 | 0,02 |
| **EC1** | **D257G** | c.770A>G | Sporadic | -1,93 | -2,21 | 0 |
| **EC1** | **P260L** | c.779C>T | Sporadic | 3,71 | 6,38 | 0 |
| **EC2** | **S270A** | c.808T>G | Sporadic | -0,02 | -0,07 | 0,02 |
| **EC2** | **M282I** | c.846G>A | Sporadic | 2,19 | 1,97 | 0,08 |
| **EC2** | **A286T** | c.856G>A | Sporadic | 4,47 | 6,8 | 1 |
| **EC2** | **T295I** | c.884C>T | Sporadic | -0,07 | 2,03 | 0,01 |
| **EC2** | **A298T** | c.892G>A | HDGC | 8,31 | 4,31 | 0,01 |
| **EC2** | **A298D** | c.893C>A | Sporadic | 11,35 | 7,78 | 0,1 |
| **EC2** | **I300M** | c.900C>G | Sporadic | 0,01 | 0,7 | 0,02 |
| **EC2** | **D308N** | c.922G>A | Sporadic | 0,13 | 0,07 | 0,31 |
| **EC2** | **P309S** | c.925C>T | Sporadic | -0,97 | 4,61 | 0,01 |
| **EC2** | **L311F** | c.931C>T | Sporadic | -0,2 | 0,03 | 0,05 |
| **EC2** | **N315S** | c.944A>G | Sporadic | -0,25 | 0,08 | 0,11 |
| **EC2** | **T330I** | c.989C>T | Sporadic | -0,23 | 0,61 | 0,01 |
| **EC2** | **D334H** | c.1000G>C | Sporadic | 1,23 | 2,1 | 0 |
| **EC2** | **P339L** | c.1016C>T | Sporadic | 1,37 | 0,79 | 0,26 |
| **EC2** | **T340A** | c.1018A>G | HDGC/Sporadic | 1,09 | 0,9 | 0,08 |
| **EC2** | **T340M** | c.1019C>T | Sporadic | -0,42 | 0,16 | 0,01 |
| **EC2** | **V344M** | c.1030G>A | Sporadic | -0,34 | -0,39 | 0,1 |
| **EC2** | **A347V** | c.1040C>T | Sporadic | 0,22 | 1,24 | 0 |
| **EC2** | **E353K** | c.1057G>A | Sporadic | -0,69 | -0,41 | 0,52 |
| **EC2** | **T357I** | c.1070C>T | Sporadic | -0,22 | 1,21 | 0,01 |
| **EC2** | **V365L** | c.1093G>C | Sporadic | 0,54 | 3,94 | 0,08 |
| **EC2** | **N369D** | c.1105A>G | Sporadic | 5,13 | 5 | 0 |
| **EC2** | **D370H** | c.1108G>C | Sporadic | 1,82 | -0,93 | 0 |
| **EC2** | **D370A** | c.1109A>C | Sporadic | -0,98 | -1,53 | 0 |
| **EC2** | **P373L** | c.1118C>T | HDGC | 1,12 | 5,02 | 0 |
| **EC3** | **P385L** | c.1154C>T | Sporadic | -0,45 | 1,13 | 0,01 |
| **EC3** | **E386K** | c.1156G>A | Sporadic | 3,81 | 1,17 | 0 |
| **EC3** | **T399I** | c.1196C>T | Sporadic | 0,19 | -0,15 | 0,01 |
| **EC3** | **D400Y** | c.1198G>T | Sporadic | 6,64 | 14,18 | 0 |
| **EC3** | **A401T** | c.1201G>A | Sporadic | 1,06 | 0,87 | 0,42 |
| **EC3** | **D402N** | c.1204G>A | Sporadic | -1,8 | -1,47 | 0 |
| **EC3** | **W409R** | c.1349T>C | HDGC | -0,24 | -0,31 | 0 |
| **EC3** | **T414I** | c.1241C>T | Sporadic | 0,12 | 0,06 | 0,03 |
| **EC3** | **I415L** | c.1243A>C | HDGC | -0,9 | 0,18 | 0,01 |
| **EC3** | **P429S** | c.1285C>T | HDGC | 1,56 | 1,89 | 0,02 |
| **EC3** | **V456M** | c.1366G>A | Sporadic | 0,9 | 6,06 | 0,01 |
| **EC3** | **E463Q** | c.1387G>C | Sporadic | -0,72 | -0,51 | 0,09 |
| **EC3** | **V473D** | c.1418T>A | Sporadic | 2,95 | 3,57 | 0 |
| **EC3** | **D479G** | c.1436A>G | Sporadic | -1,23 | -0,53 | 0,01 |
| **EC4** | **V487A** | c.1460T>C | HDGC | 0,45 | 0,39 | 0,56 |
| **EC4** | **S559N** | c.1676G>A | Sporadic | -0,05 | 1,13 | 1 |
| **EC4** | **G571S** | c.1711G>A | Sporadic | 8,34 | 2,28 | 0,01 |
| **EC4** | **L581P** | c.1742T>C | Sporadic | 9,51 | 5,08 | 0 |
| **EC4** | **A592T§** | c.1774G>A | HDGC/Sporadic | 3,42 | *-0,72* | 0 |
| **EC5** | **R598Q** | c.1793G>A | Sporadic | 0,65 | *0,12* | 0,59 |
| **EC5** | **T599S** | c.1796C>G | HDGC | 0,22 | *-0,21* | 0,2 |
| **EC5** | **I600M** | c.1800A>G | Sporadic | -2,93 | *-2,61* | 0,23 |
| **EC5** | **A617T§** | c.1849G>A | HDGC/Sporadic | 0,06 | -0,4 | 0,43 |
| **EC5** | **F626V** | c.1876T>G | Sporadic | 2,48 | *2,19* | 0 |
| **EC5** | **A634V*** | c.1901C>T | HDGC/Sporadic | 1,05 | *3,59* | 0,26 |
| **EC5** | **A692V** | c.2075C>T | Sporadic | 0,12 | N/A | 0,64 |
| **CBD** | **P799R** | c.2396C>G | HDGC | 0,62 | - | 0,03 |
| **CBD** | **V832M** | c.2494G>A | HDGC | -1,1 | - | 0 |
| **CBD** | **S838G** | c.2512A>G | Sporadic | -0,88 | - | 0,18 |
| **EC1** | **E185V** (a) | c.554A>T | HDGC | 0,29 | 0,29 | 0,25 |
| **EC1** | **S232C** (a) | c.695C>G | HDGC | -0,9 | -0,85 | 0,01 |
| **EC4** | **L583R** | c.1748T>G | HDGC | 2,72 | 5,39 | 0 |
| **EC4** | **L583I** (a) | c.1747C>A  c.1749G>T | Artificial | 1,41 | 0,56 | 1 |

Structural impact is considered when ΔΔG>0,8kcal/mol in the FoldX column, and values bellow 0,05 in the SIFT column are considered to be intolerant due to high conservation. Newly identified HDGC-associated mutations are listed on the bottom of the table, with unpublished marked with (a). **ΔΔG_model_** is the stability change as calculated in the models described in Material and Methods; **ΔΔG_mouse_** contains the results when the calculations are made in Chain B of a recent PDB annotated for mouse E-cadherin extracellular domain (3Q2V), using a similar method. Italic is used to distinguish the values calculated in Chain A, due to lack of coverage in Chain B. HDGC, Hereditary Diffuse Gastric Cancer; CBD – Cadherin Binding Domain.
